# Development and initial validation of MedHipPro-Q: a questionnaire assessing medication management of hip fracture patients in different care settings

**DOI:** 10.1186/s12913-022-07524-2

**Published:** 2022-02-22

**Authors:** Ben Tore Henriksen, Yvonne Andersson, Maren Nordsveen Davies, Liv Mathiesen, Maria Krogseth, Randi Dovland Andersen

**Affiliations:** 1 Tonsberg Hospital Pharmacy, Hospital Pharmacies Enterprise, South-Eastern Norway, Tonsberg, Norway; 2 Vestfold Hospital Trust, Tonsberg, Norway; 3 Department of Pharmacy, The Faculty of Mathematics and Natural Sciences, University of Oslo, Oslo, Norway; 4 Hospital Pharmacies Enterprise, South-Eastern Norway, Oslo, Norway; 5 Department of Internal Medicine, Telemark Hospital Trust, Skien, Norway; 6 Old Age Psychiatry Research Network, Telemark Hospital Trust and Vestfold Hospital Trust, Tonsberg, Norway; 7 University of South-Eastern Norway, Drammen, Norway; 8 Department of Research, Telemark Hospital Trust, Skien, Norway; 9 Research Centre for Habilitation and Rehabilitation Models & Services (CHARM), The Faculty of Medicine, University of Oslo, Oslo, Norway

## Abstract

**Background:**

A validated questionnaire to assess medication management of hip fracture patients within and outside the hospital setting was lacking. The study aims were to describe the hip fracture patient pathway, and develop a valid and feasible questionnaire to assess clinicians’ experience with medication management of hip fracture patients in different care settings throughout the patient pathway.

**Methods:**

This qualitative, descriptive methodological study used strategic and snowball sampling. The questionnaire was developed, and face and content validity explored through interviews with stakeholders. Phase I described the hip fracture patient pathway, and identified questionnaire dimensions in semi-structured interviews with management and clinicians (*n* = 37). The patient pathway was also discussed in six meetings (*n* = 70). Phase II refined a first draft of the questionnaire through cognitive interviews with future respondents (*n* = 23). The draft was modified after each interview. Post hoc, cognitive interview data were analysed using matrix analysis to condense problems and solutions into themes and subthemes. Phase III, converted the final version to a digital format, and tested its feasibility with a subset of the cognitive interview participants (*n* = 21) who completed the questionnaire and provided feedback.

**Results:**

Phase I: Hip fracture patients were cared for in at least three different care settings, and went through at least four handovers between and within primary and secondary care. Three questionnaire dimensions were identified: 1) Medication reconciliation and review, 2) Communication of key information, and 3) Profession and setting. Phase II: The MedHipPro-Q was representative of how the different professions experienced medication management in all settings, and hence showed face and content validity. Post hoc analysis: Problem themes (with sub-themes) were *Representativeness* (*-of patient pathway* and -*of respondent reality*) and *Presentation* (*Language* and *Appearance*)*.* Solution themes (with sub-themes) were: *Content* (*added* or *deleted*) and *Presentation* (*modified appearance* or *corrected language*). Phase III: Participants did not identify technical, linguistic or content flaws in the questionnaire, and the digital version was considered feasible for use.

**Conclusion:**

The novel MedHipPro-Q showed good face and content validity, and was feasible for use throughout the hip fracture patient pathway. The rigorous development process supports its construct validity and reliability.

**Supplementary Information:**

The online version contains supplementary material available at 10.1186/s12913-022-07524-2.

## Background

Risk of disease increases with age, and older persons frequently suffer from more than one chronic condition that necessitates pharmacological treatment. Consequently, polypharmacy, often defined as the use of five or more regular medications, is common in older patients [1]. Also, older persons are more often hospitalised, and hence having multiple care settings involved in medication management (e.g. prescribing and administration) [2]. Hip fracture patients are characterised by a high mean age [3], they suffer from comorbidities [4, 5], and consequently polypharmacy is common [6]. In addition, hip fractures necessitates surgery and rehabilitation, and thus transfer within and between care settings. Care transitions increases the risk of discrepancies in medication lists [7]. Such discrepancies constitutes a potential risk to patient safety as it may lead to adverse events, such as inadequate treatment or (re)hospitalisation, and, in the most extreme cases, permanent harm [8]. Clear communication between care settings is thus of major importance but is often deemed inadequate [9]. To prevent medication discrepancies after transfer between care settings, medication reconciliation, i.e. identification of an accurate list of current medicines, is considered fundamental [2, 10]. In addition to the need for a correct medication list (i.e. medication reconciliation has been performed), polypharmacy manifests a need for medication review to assure optimal treatment and prevent unwanted side effects [11]. The existing studies on transfer of hip fracture patients between primary and secondary care [12, 13] do not focus on medication management (i.e. prescribing of medication, its administration, reconciliation, review).

Due to the patient safety risks described above, there was a need for improving medication management, and secure safe care transitions for the hip fracture patient within the hospital fast track and primary care settings. To plan a clinical intervention to improve hip fracture patient care, we needed a tool to map the current situation in regard to medication management, identify areas for improvement, and with the potential to measure any changes in medication management. Using a previously developed and validated questionnaire would be preferable. However, although questionnaires to evaluate clinicians’ experiences for example in the operating theatre [14], and in regard to attitudes to patient safety [15] existed, we were not able to identify a suitable questionnaire for our purpose. A questionnaire as described above, would be useful for future research or clinical quality assurance projects addressing medication management.

Consequently, we decided to develop a new questionnaire to assess clinicians’ experience with medication management of hip fracture patients across different care settings (the MedHipPro-Q).

## Methods

The aims of this study were to describe the hip fracture patient pathway, and develop a valid and feasible questionnaire to assess clinicians’ experience with medication management of hip fracture patients throughout the patient pathway (the MedHipPro-Q).

### Study design

**Fig. 1:**
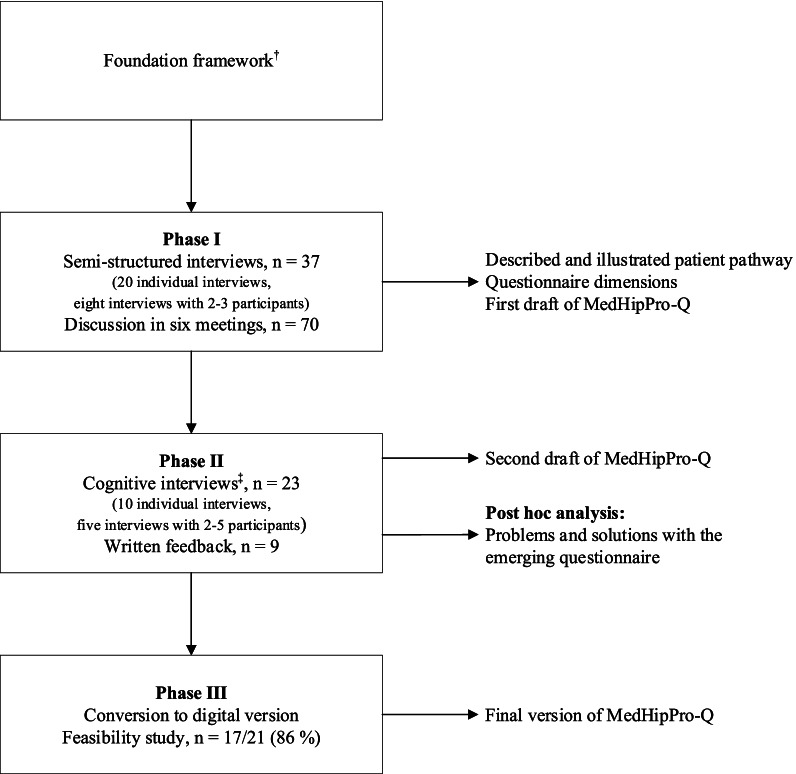
Study flow diagram for the development of The MedHipPro-Q. *n* = participants, ^†^The foundation framework implies the Norwegian patient safety programme [16], the in-hospital hip fracture fast track, and Integrated Medicines Management-method [17–20]. ^‡^Phase II used a dynamic approach where the draft questionnaire was modified after each interview, and the adapted version was given the following interviewee

This descriptive methodological study used a qualitative approach and was conducted in three phases (Fig. 1). The foundation framework for the questionnaire development process consisted of the Norwegian patient safety programme [16], the in-hospital hip fracture fast track, and Integrated Medicines Management-method [17–19]. The questionnaire was developed, and face and content validity was explored through interviews with stakeholders. First, the hip fracture patient pathway was described, questionnaire dimensions identified, and the initial version of the questionnaire developed. In this study, the term ‘patient pathway’ refers to the hip fracture patient’s journey across all care settings; from fracture, through hospital fast track, post-discharge rehabilitation, and lastly back to their permanent residence. Second, the questionnaire was further developed and refined, and finally, a feasibility study of the digitalised questionnaire was performed.

The study is reported in accordance with the COnsolidated criteria for REeporting Qualitative research (COREQ) guideline [21].

### Ethics approval and consent to participate

The study did not to meet the requirements for medical and health research, cf. Act on medical and health research (the Health Research Act), Stat. ACT-2008-06-20. No. 44. Norwegian Ministry of Health and Care Services (2008). According to the existing Norwegian Law in force prior to General Data Protection Regulation (GDPR) it was not necessary to seek privacy protection approval due to the inherent anonymity, and on the basis of voluntarily contribution from leaders and healthcare personnel without recordings, verbatim transcripts, or any other identifiable material, cf. the Personal Data Act, Stat. ACT-2000-04-14. No 31. Norwegian Ministry of Justice and Public Security (2000). Written consent for this study was deemed unnecessary according to hospital’s internal legislation and national regulations, cf. Act on medical and health research (the Health Research Act), Stat. ACT-2008-06-20. No. 44. Norwegian Ministry of Health and Care Services (2008), and the Personal Data Act, Stat. ACT-2000-04-14. No 31. Norwegian Ministry of Justice and Public Security (2000).

### Setting and sample

The study took place in a region in South-Eastern Norway (approximate population: 250 000) from September 2017 to August 2018. All hip fracture patients from surrounding local authorities are admitted to the regional hospital (Vestfold Hospital Trust; VHT, approximately 5200 employees and 550 beds) and entered into the in-hospital hip fracture fast track. The relevant care settings were: the emergency care unit (secondary care), orthopaedic department (secondary care), nursing home/rehabilitation institution (primary care), and home-dwelling setting (primary care).

### Samples

Eligible participants were management (leaders and advisors) and clinicians (i.e. physicians and nurses) involved in hip fracture patient care within the region.

#### Sample I

A snowball sampling strategy [22] was used to recruit participants (*n* = 37) for the phase I interviews. The sampling started at the regional hospital with a director who was familiar with the hospital fast track. Participants were selected to cover all settings of the emerging patient pathway from the perspectives of management and clinicians. If any setting was not represented, we sought advice from previously included participants to identify and recruit relevant personnel from the missing setting.

The emerging hip fracture patient pathway was also discussed in six meetings with 70 participants in total. Participants at the meetings were management and clinicians involved in hip fracture patient care within the region, in addition to non-regional clinicians, pharmacists and leaders with special interest in medication management.

#### Sample II

Leaders were asked to recruit experienced clinicians working in their setting, i.e. future questionnaire respondents (*n* = 23) for the cognitive phase II interviews. Each care setting was represented with two to four clinicians. Home-dwelling were represented with district nurses and General Practitioners (GP).

#### Sample III

A subset of sample II with two or three clinicians from each care setting were invited and 17 agreed to participate, giving a response rate of 86% (17 out of 21). Two clinicians from primary care were not invited as the care setting was already represented with the maximum number of participants.

### Data collection and preliminary analysis

Data were collected in individual or small group semi-structured interviews, or from written feedback provided by the participants. All interviews were conducted by the first author (BTH), a clinical pharmacist at VHT at the time of the study. BTH had prior experience with semi-structured interviews and questionnaire development [23]. Interview participants chose the place and time for the interview. Most interviews were conducted at the participants' workplace whilst a few were conducted at the interviewer's office. Participants had no former relation with the interviewer.

The data material consisted of field-notes and written questionnaire amendments made by the participants. The interviews were not recorded.

#### Phase I: The patient pathway and questionnaire dimensions

Due to the medication focus in the planned questionnaire, the hip fracture patient’s journey through the healthcare system needed firstly to be described with details regarding prescribing and administration responsibility.

Meetings and semi-structured interviews were conducted to establish and illustrate the patient pathway, to explore and confirm questionnaire dimensions, and to develop a first draft of the questionnaire.

The interview process was dynamic and interactive, starting with the predefined in-hospital fast track for hip fracture patients. This was expanded to describe the hip fracture patient journey from injury, through the healthcare system, and until the patient returned to their permanent residence. For each care setting, we identified clinicians’ specific medication management responsibility, including communication with care providers in other care settings. In subsequent interviews, the current draft of the patient pathway and emerging dimensions for the questionnaire were further refined based on feedback from the participants.

Phase I was concluded after saturation in feedback had been achieved. Based on the final description of the hip fracture patient pathway and the identified questionnaire dimensions, a first draft of the questionnaire was subsequently developed.

#### Phase II: Refinement of the questionnaire and initial validation

Cognitive interviews were conducted using a dynamic approach [24] to improve the questionnaire (supplementary file 1). The questionnaire draft was sent to participants 1–4 days prior to the interview, and they were asked to read through it in preparation for the interview. During the interview, information about how the participant understood questionnaire items and response options were obtained. Spontaneous comments or questions from participants were answered, and different approaches to avoid future uncertainty were investigated. Participant hesitation was followed with probes that investigated the reason for the hesitation, and how it could be avoided. If participants answered without spontaneous questions or hesitation, verbal probes were used to explore how the participant arrived at their answer. The aim was to verify that content was representative of participant reality and being interpreted as intended. If it was not, optimal amendments were identified. Participants were also asked if any aspect of the medication management for hip fracture patients were missing from the questionnaire, or if the participant were not given the opportunity to provide the feedback they wished. Alterations to the questionnaire were made either during or immediately after each interview, resulting in a new draft before the next interview. All modifications to items were allowed without any predetermined constraints [25]. Phase II ended after saturation in feedback [26].

##### Post hoc analysis of data from cognitive interviews (phase II)

We analysed the cognitive interviews post hoc to reveal patterns in the problems and solutions that were identified. A matrix was used to analyse data [27]. The rows in the matrix included 1) the content (e.g. questionnaire item, response option) prior to the interview, 2) problems identified by the participant, 3) problems identified by the researcher, 4) solution strategies, and 5) the refined content (e.g. questionnaire item or response option after changes). First, a ‘single case’ analysis was performed for each interview (individual or small group) or written feedback. All content that received comments or amendments were included and delegated a separate row in the matrix. After single case-analysis was performed for all cases, a cross-case analysis was performed using thematic reduction to condense the material into themes with subthemes for problems and solutions. Problems identified by the participant, problems identified by the researcher, and the solutions applied were analysed separately. The problems identified by the participant and by the researcher were subsequently combined.

The matrix analysis was performed using Microsoft® Office Excel®, 2016.

#### Phase III: Feasibility

The questionnaire was adapted to a digital format (Questback®, 2018 [28]) by an adviser with special competence in the chosen digital questionnaire platform. Feasibility was tested by participants who completed the digital version of MedHipPro-Q and provided feedback on its feasibility, including technical guiding throughout the questionnaire, linguistic or content flaws, and the time it took to complete the questionnaire.

#### Translation

The MedHipPro-Q was translated into English for publication purposes by BTH and proofread by MND, who are both bilingual and have lived in both the UK and in Norway. Consensus was achieved after discussion if any improvements were suggested.

## Results

### Phase I: Patient pathway and questionnaire dimensions

**Fig. 2:**
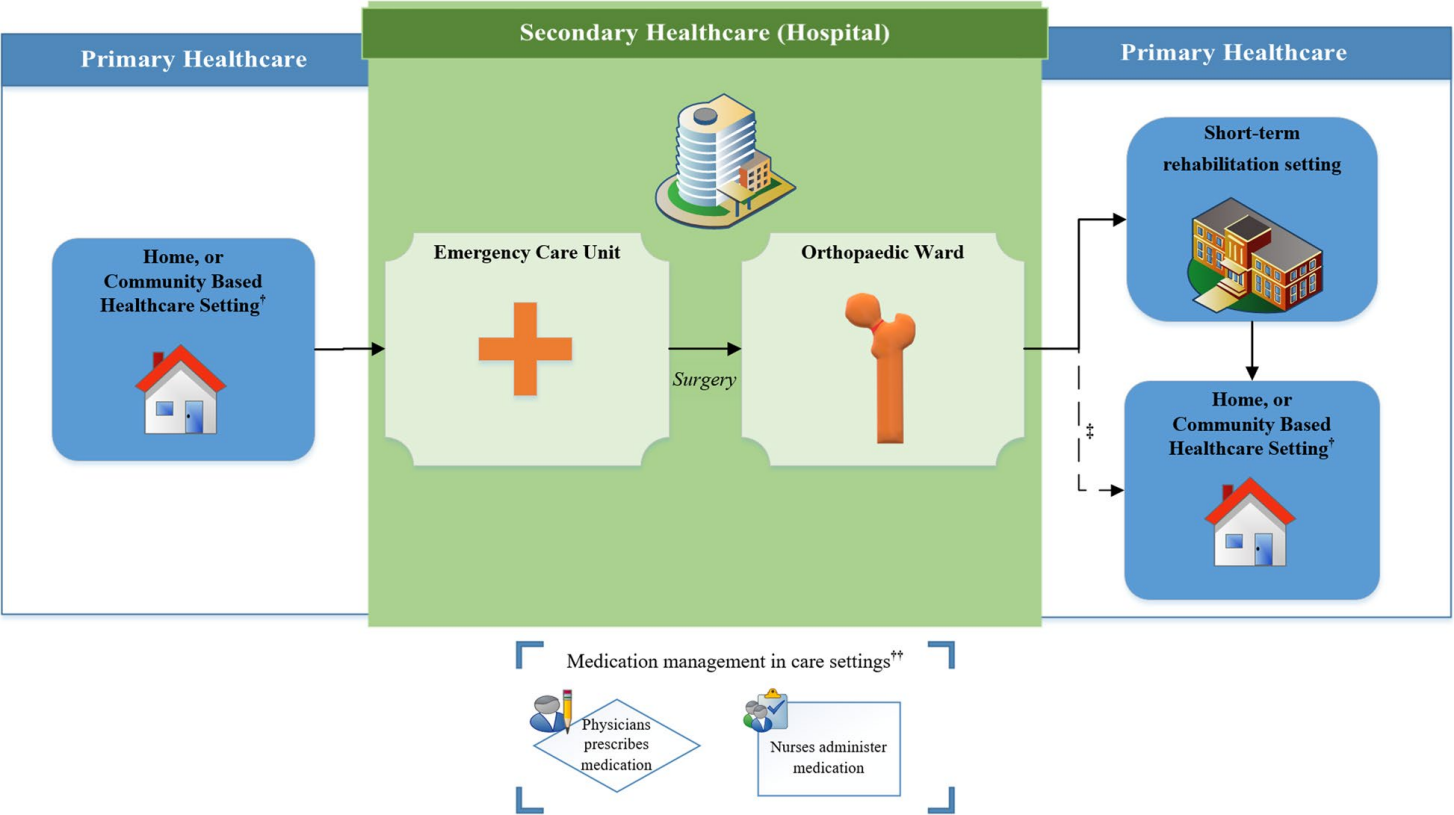
Hip Fracture Patient Pathway in the Norwegian Healthcare System from a medication perspective. ^†^Patients who had an increased need for care received community based healthcare in one of the following care settings: Home with district nursing service, Care home with an increased follow-up by the district nursing service, or Nursing home—where the patient had a 24-h nursing service with affiliated physicians available at least once a week. The healthcare personnel responsible for prescribing and administering medications were affiliated with the accommodation setting (e.g. if in a nursing home; the physician at the nursing home prescribed medication, and nursing staff administered). ††Medication management in care settings: physicians were responsible for prescribing medication (e.g. General Practitioner for home-dwelling patients, the affiliated physician for patients in nursing homes), and nurses were responsible for the administration of medication. For home-dwelling patients, the patients themselves, a carer, or district nurses were responsible for administration. ‡Some patients were discharged directly to their habitual accommodation. Patients without adequate support from community based healthcare received increased care for a limited time period, whilst recovering, or if needed; permanently

Based on the established in-hospital fast track, and findings from the interviews, we described the hip fracture patient pathway with an emphasis on medication management (Fig. 2).

Three questionnaire dimensions were determined:

1. *Medication reconciliation and review* covered how medication review was performed and to what extent (e.g. number of patients and frequency).
2. *Communication of key information* covered the transfer of medication list and treatment plan between care settings. An important aspect was how to assure the medication list’s quality.
3. *Profession and setting* addressed the respondents in the patient pathway, their qualifications, experience, and their medication management tasks related to the specific setting in which they worked.

### Phase II: Refinement of the questionnaire and initial validation

During the cognitive interviews several problems with the questionnaire were discovered and corrected. In the post hoc analysis, we identified that problems and solutions each formed two separate themes.

#### Problem themes

Identified problems formed the two themes – *Representativeness* – i.e. issues with questionnaire content, and – *Presentation* – i.e. issues with how the questionnaire content was presented to or perceived by the participant (Table 1).

**Table 1:** Problem themes and subthemes identified through the cognitive interviews

Theme	Subtheme	Categories
**Representativeness**	Representative of patient pathway	Missing or superfluous content
		Relevant content not assigned to a respondent group
	Representative of participant reality	Too generic or unsuitable content
		Lacking information
**Presentation**	Language	Proofreading error
		Ambiguous terms and expressions
		Complicated syntax
		Passive voice
	Appearance	Inadequately presented information
		Redundant content
		Construction and structure
		Insufficient support information
		Guiding issues
		Layout and formatting

##### Representativeness

The theme Representativeness included problems with how well the questionnaire content reflected the patient pathway as described in phase I – *Representative of patient pathway* – or the participant’s opinion of how patient care and medication management were carried out in their clinical practice – *Representative of participant reality* –. Thus, these two subthemes reflected the two angles from which the questionnaire was challenged.

The first sub-theme—*Representative of patient pathway* – comprised missing or superfluous content and respondent groups not being assigned relevant content.

Key aspects of the patient pathway were not sufficiently covered by the emerging questionnaire, because items, or response options were missing. To illustrate, the dimension ‘medication reconciliation and review’, missed three aspects: 1) who performed the medication review, 2) the opportunity to perform medication reviews more frequently than currently possible for the clinician, and 3) situations where the medication review might be performed by someone else than the participant.

In other instances, an existing item was not made available to a participant it was relevant for. Problems in this category usually emerged after a verbal probe by the interviewer (e.g. ‘this question is assigned to your profession, but is it relevant to you?’). An example was items regarding the medication list in discharge summaries. These questions were initially directed towards GP’s only, but we found that they were also relevant for other clinicians in primary care.

“Questions regarding medication list in discharge summaries are relevant for physicians in nursing homes/rehabilitation institutions, but the questions are for GP’s only” (P1)

Contrastingly, administration of irrelevant content to participants was also revealed.

“As the questionnaire content is currently presented, all physicians are assigned questionnaire items regarding medication review. However, not all physicians perform medication review.” (P11-14)

The second sub-theme – *Representative of participant reality* – comprised content being perceived as too generic, not being relevant for the participant’s work situation, or the participant lacked the information necessary to understand the content as intended.

Participants frequently identified questionnaire content that did not fit their clinical practice, like this example regarding transfer of patients:

“From the orthopaedic department’s point-of-view, the patients are transferred from the emergency room, not directly from primary care as the questionnaire item now insinuate” (P7-8)

Participants also identified wording that did not correspond with the language used in their clinical practice:

“Physicians at the rehabilitation institution use the term ‘discharge report’, not ‘discharge summary’” (P16-17)

Furthermore, the participants needed the questionnaire to provide enough information for them to interpret the content as intended. For example, we discovered that it was difficult to picture relevant clinical scenarios when responding to the questionnaire (P11-14).

##### Presentation

Presentation comprised problems with content not being understood as intended, included in the sub-theme – *Language* –, and problems related to how the questionnaire was constructed, structured, and its layout, included in the sub-theme – *Appearance* –.

The first sub-theme – *Language* – comprised problems with language ranging from simple proofreading errors and misspelling, to participant comprehension issues due to complicated syntax, and the use of passive voice.

Participants experienced ambiguous terms as a problem. For example, the term ‘medication reconciliation’ (P1) or the term ‘sources’ (P18-20). In context to medication reconciliation, ‘sources’ implies from whom or where the medication lists were derived, but this term was difficult to interpret.

Participants also identified complicated syntax that made it difficult to understand items or response options. Lastly, participants found that the use of passive voice was problematic. According to one participant passive wording made a statement appear less relevant (P9).

The second sub-theme – *Appearance* – comprised problems related to: inadequately presented information, redundant content, construction and structure, insufficient support information, guiding issues, and layout and formatting.

Information was sometimes perceived as inadequately presented. For instance, in some of the earlier interviews (P7-8), the researcher had to remind the participants that the questionnaire asked about hip fracture patients only, even though this information was present in the questionnaire.

All information that was not directly relevant for the participant or required to respond to the questionnaire was problematic, as it increased the length of the questionnaire, competed with key information and increased respondent burden.

Participants also helped identify sub-optimal organisation of the questionnaire. In an earlier draft of the questionnaire, some items were not organised within their respective dimensions. Instead, a set of statements addressing different dimensions were organised together as a series of statements to be answered using Likert-scale response options. One participant commented:

“It is difficult to grasp and complicates following the order of questions when these statements are in a separate questionnaire section” (P15)

Some of the participants found missing support information problematic, for example regarding incentives to participate (P25). Both participants and the researcher identified incorrect guiding and skipping patterns, inconsistent text type, size and/or colour.

#### Solution themes

The solutions formed the two themes – *Content* – i.e. adding or deleting content, and – *Presentation* – i.e. adjusting the presentation of content already present (Table 2).

**Table 2:** Solution themes and subthemes identified through the cognitive interviews

Theme	Subtheme	Categories
**Content**	Added content	Added definition of key terms and phrases
		Added questionnaire item / response option
		Assigned content to a new respondent group
		Added prompt or assisting text
	Deleted content	Removed superfluous questionnaire item or response option
		Removed content not relevant to the respondent group in question
		Removed unnecessary text
**Presentation**	Modified appearance	Rearranged content within the questionnaire
		Changed layout or formatting
		Corrected guiding
	Corrected language	Simplified terms, phrases or syntax
		Rewrote text in active voice
		Adjusted terms and phrases to better represent the participant’s reality

##### Content

The solution theme – *Content* – comprised the sub-themes – *Added content* – i.e. introduced new content, and – *Deleted content* – i.e. removed content that was already present.

*Added content* included introducing definitions of key terms and phrases, new questionnaire items and response options, and assigning content to a new respondent group. An example of added content was the introduction of scenarios (added assisting text) to illustrate which patients to include, and which context to picture (P11-14). The scenarios were tailored for each healthcare profession, at each setting, to provide enough information for participants to interpret content as intended.

The second sub-theme *Deleted content* comprised the removal of superfluous questionnaire items or response options, content not relevant to the respondent group, and unnecessary text. An example of a superfluous questionnaire item was “I would save time if I could always trust that the medication list I receive is correct”. The response to this item was perceived as a truism, and the item was deleted (P21).

##### Presentation

The theme – *Presentation* – comprised solutions that adjusted content already present in the questionnaire through – *Modified appearance* –, and – *Corrected language* –. Modifying appearance was applied by rearranging content within the questionnaire, changing layout or formatting (e.g. text size, colours), or correcting guiding patterns. If questionnaire content needed linguistic improvements, we corrected the language by simplifying terms, phrases or syntax, rewriting text in active voice, or adjusting terms and phrases to better represent the participant’s reality. To illustrate, the item «When a medication review is performed, is the medication review documented?» was changed by simplifying the syntax to «Are medication reviews documented?» (P7-9).

#### Connecting problems and solutions

Some problems had logical solutions, such as introducing missing content (e.g. questionnaire items), or correcting flawed skipping patterns. Other problems differed more in solution strategies. An example was complicated syntax, a problem which could be solved by simplifying the syntax, or by deleting the item or response option if simplification was infeasible. The problem category with the most diverse solution strategies was *Ambiguous terms and phrases*. Problems in this category could be solved by adding definitions of key terms and phrases, changing the terms and phrases used, introducing text to facilitate a mental image of a relevant real-life situation for respondents, or introducing a condition or context to the phrase to narrow possible interpretations of it. The solution to irrelevant content was often to remove it, but other solution strategies was also applied. To illustrate the latter, items regarding medication reviews were administered to all physicians, however, not all physicians performed medication reviews. For this problem, we added an introductory item asking if the respondent performed medication reviews. If the answer was no, the irrelevant content was skipped.

Sometimes a single problem required more than one solution. For instance where a participant explained that nurses in the Emergency Care Unit were not responsible for medication reconciliation (P21). The solution was firstly to remove medication reconciliation items from the questionnaire version administered to these nurses and secondly to introduce seven new questionnaire items for them, resulting in a new subsection. The items connected to medication reconciliation influenced all items in the new subsection. Lastly, the new questionnaire items needed further refinement.

The final version of the MedHipPro-Q (Supplementary file 2).

### Phase III: Feasibility

The feasibility study did not identify any technical flaws in the digital questionnaire and no changes were made. Time for completion was estimated to 9 min (range: 5–14 min).

## Discussion

The main finding in the present study was that the MedHipPro-Q showed good feasibility, face and content validity. The hip fracture patient pathway included three to five different care settings. The patient went through at least two handovers between primary and secondary care, two handovers within secondary care, and usually two handovers within primary care. To assess clinicians’ experiences with the medication management of hip fracture patients throughout the pathway, three questionnaire dimensions were identified: 1) Medication reconciliation and review, 2) Communication of key information, and 3) Profession and setting. The MedHipPro-Q was subsequently developed, and, during a series of cognitive interviews, tailored to clinicians working in five care settings throughout the pathway. Analysis of these interviews revealed that problems with the emerging questionnaire formed the two themes “Representativeness” and “Presentation”, whilst their solutions formed the two themes “Content” and “Presentation”.

Hip fracture patients were cared for in multiple settings and went through several handovers. All transfers between care settings involve a pivotal transfer of key information, including a medication list and a treatment plan, and increase the risk for medication errors and subsequent harm to the patient [2, 29]. A key difference between previous research and this study, was that it addressed patient care and transfers between different care settings throughout the entire patient pathway, including primary care. Prior studies on the other hand, have primarily focused on in-hospital care and/or hospital discharge [12, 13, 30–32]. As a consequence, the MedHipPro-Q has a greater potential for identifying problems related to medication management, and subsequent improvement of patient safety than studies focusing only on in-hospital care and discharge.

The MedHipPro-Q needed to cover how medication reconciliation and review were performed, and to what extent (i.e. how many patients that has a correct medication list and receives medication review), as stakeholders during phase I-interviews described it in varying degree. Having ‘Medication reconciliation and review’ as a dimension in the questionnaire was consistent with the importance of these procedures for patients with polypharmacy [7–11]. Some aspects in this dimension, such as the number of patients arriving with a correct medication list, are also found in a questionnaire assessing medication reconciliation in an emergency care unit and community pharmacies [33]. However, one advantage of the MedHipPro-Q compared to existing questionnaires, was its coverage of the entire patient pathway, compared to the two specific settings only. We believe the questionnaire items in this dimension will provide useful information on whether these tasks are being successfully completed, or if medication reconciliation and review are areas that may profit from specific actions to improve healthcare service delivery.

Participants experienced care transitions as a particularly weak link in the patient pathway. Thus, the questionnaire dimension ‘Communication of key information’ included items related to transfer of information regarding pharmacotherapy. These findings were consistent with national and international focus on improvement of medication management [2, 9, 16], and previous literature [29, 34–36] which explains that communicating information in patient handovers related to medications is a threat to patient safety if the information is not of high quality. The MedHipPro-Q may be used to detect weak links in care transitions, and identify specific actions that may improve patient care.

In our opinion, the development approach we have described, and the themes that emerged supported the face and content validity of the MedHipPro-Q. An indication of the content validity is shown from how the inductively formed themes aligned with questionnaire literature. The results showed that it was important that the MedHipPro-Q were representative of both the patient pathway and participant reality. This is consistent with previous studies that describe how questionnaires need to represent the phenomenon it investigates [24, 37]. The participants also highlighted the importance of receiving relevantly worded questions and response options, as well as not being distributed items that were not relevant for them [24, 38–41].

We found that participants needed a common understanding of questionnaire content, and that they were able to give an accurate response to items. This robustness against misinterpretation was a significant focus for improvement during the cognitive interviews. Questionnaire reliability concerns its stability, i.e. yielding the same response under the same conditions, on separate occasions [42]. The most widespread reliability testing approach is quantitative measures, such as factor analysis to identify dimensions of the questionnaire and Cronbach’s alpha for internal consistency [43]. However, supporting reliability using a qualitative approach has been described previously [39]. We would argue that a common interpretation of items and provision of responses across respondents, and being in accordance with the questionnaire developer’s intentions, supports the reliability of the questionnaire.

We would further argue that the qualitative approach used also contributed to construct validity of the MedHipPro-Q as a key aspect of construct validity is to what extent a questionnaire is representative of the phenomenon it is designed to investigate. The inclusion of a diversity of stakeholders provided good understanding of the patient pathway and questionnaire content, and the subsequent cognitive interviews included potential future respondents who were experts on hip fracture patient care in their particular setting, thus capturing problems perceived by the participants and resolved them. These problems would not have been detected if we proceeded directly to psychometric testing, which has been described previously as a concern [39, 40].

In the post-hoc analysis, we identified problems related to the questionnaire’s representativeness, and the mental processing of questionnaire items and response options. We followed recommendations on conducting cognitive interviews to ensure clear wording to support the content validity of the questionnaire [24, 39, 40]. Conrad and Blair’s Problem Solving Matrix (PSM) [44] was initially applied deductively in the post-hoc analysis to categorise problems in the cross-case, but only fitted problems related to participants’ answering process. Many problems found in phase II fell outside their scope of problems, for example whether or not a questionnaire item should be included or other problems related to *Representativeness*. We found no applicable alternatives to the PSM in the literature, hence used an inductive approach. A strength of this study was that two authors analysed the cognitive interviews (BTH and RDA), with regular feedback from a third author (YA). During the post hoc-analysis, we considered quantifying the problems in each theme. However, there is a risk of giving an incorrect impression on how important a theme or sub-theme was; a problem mentioned by only a few participants could be more important than a problem mentioned by many [26, 39, 45]. Thus, we decided to describe the problems qualitatively.

### Implications for research and clinical practice, and future studies

We believe the MedHipPro-Q will be useful for practitioners and researchers who want to improve patient care and safety by identifying problems in regards to medication management, or plan a clinical intervention to improve medication management. The MedHipPro-Q may be used as a baseline measure, and potentially to detect change over time.

Task shifting has been suggested to ease the workload of busy clinicians, enabling them to allocate more time towards their specialised responsibilities [46]. The MedHipPro-Q may be used to identify medication management issues that takes up clinicians’ time unnecessary, and assist in resolving these issues by task shifting to other healthcare personnel. For example, additional work from tasks not performed by others, or tasks that may be performed by other personnel, such as a pharmacist.

The MedHipPro-Q covers perceptions of clinicians from the entire patient pathway, involving two departments in secondary care, as well as multiple institutions in several local authorities. The questionnaire may provide useful insights by those closest to patients and the medication management tasks. Findings may be combined with more objective data from retrospective medical records review.

The MedHipPro-Q needs to be further validated, in particular in regard to its reliability, construct validity and ability to detect change over time.

Despite the need for further psychometric testing, we believe the MedHipPro-Q will be a suitable tool to investigate clinicians’ opinions on hip fracture patient care, as a part of a medication management project. A baseline description of the patient population, and to what extent medication reconciliation and review is performed, is needed as a starting point for a planned clinical pharmacist intervention. Results will be used to identify limitations in the current health services, and to inform a clinical pharmacist intervention to improve pharmacological treatment, ensure safe care transitions, and ease clinicians’ workload.

The MedHipPro-Q is ready for distribution after minor local adaptions to other Norwegian settings. The questionnaire may also be useful in countries with similar organisation of the healthcare system, but will require cultural adaptation and piloting. In particular, the specific tasks may be different in other health services, such as in the UK where there are non-medical prescribers [47]. The questionnaire dimension ‘Profession and setting’ may be adapted to fit other healthcare systems to ensure the correct content is assigned to the right respondents.

We believe both the MedHipPro-Q, this approach to questionnaire development, and the themes identified in the post hoc analysis may be useful for future studies. Although the questionnaire was developed for hip fracture patients it may serve as a template for evaluation of medication management for other patient groups. Furthermore, development of similar questionnaires for other patient groups, may be modelled after the process described in this study. The problem and solution themes identified through the post hoc analysis appear largely generic in nature, and may provide useful information for researchers developing questionnaires, but this assumption needs to be further explored.

## Conclusion

The novel MedHipPro-Q demonstrated feasibility, good face and content validity, and may be used to assess respondents’ experiences regarding hip fracture patients’ medication management throughout the entire patient pathway.

## Supplementary Information

**Additional file 1. **Cognitive interview approach of phase II in the development of the MedHipPro-Q.

**Additional file 2. **The MedHipPro Questionnaire [English paper version].

## Data Availability

The datasets used and/or analysed during the current study are available from the corresponding author on reasonable request.

## Abbreviations

GP: General Practitioner
PSM: Problem Solving Matrix
VHT: Vestfold Hospital Trust
