# Supplementary material for: Development and initial validation of MedHipPro-Q: a questionnaire assessing medication management of hip fracture patients in different care settings
Source: BMC Health Serv Res. 2022 Feb 22;22:240. doi: 10.1186/s12913-022-07524-2 (PMC8862359; doi:10.1186/s12913-022-07524-2)
Supplement: Supplementary file 1 — Additional file 1. Cognitive interview approach of phase II in the development of the MedHipPro-Q. [file 12913_2022_7524_MOESM1_ESM.pdf]

Supplementary file 1

Cognitive interview approach of phase II in the development of the MedHipPro-Q

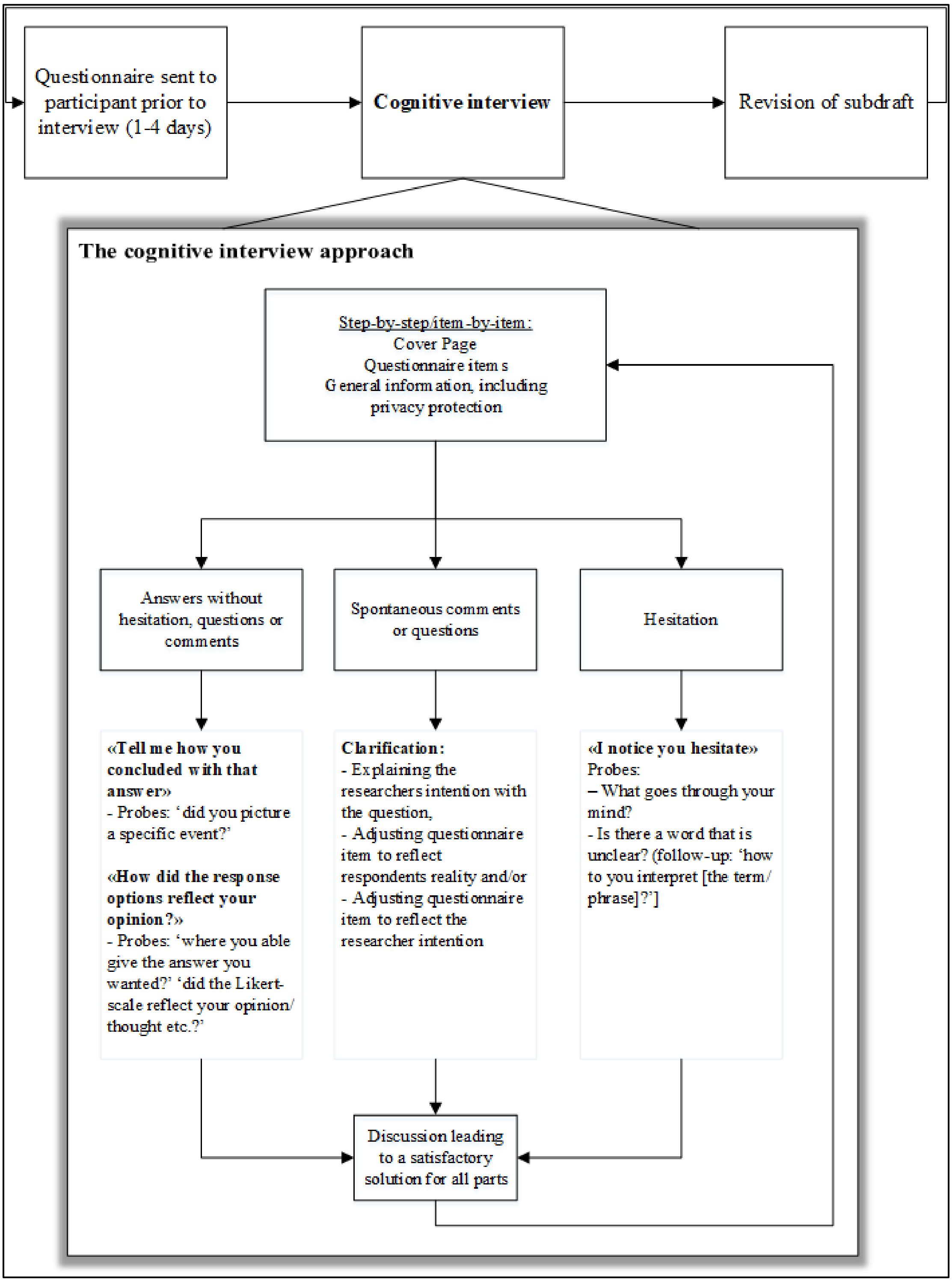

Illustration by Ben Tore Henriksen, Hospital Pharmacies Enterprise, South-Eastern Norway, as a supplementary file to the BMC Health Services Research article "Development and initial validation of MedHipPro-Q: a questionnaire assessing medication management of hip fracture patients in different care settings"
