# Supplementary material for: Development and initial validation of MedHipPro-Q: a questionnaire assessing medication management of hip fracture patients in different care settings
Source: BMC Health Serv Res. 2022 Feb 22;22:240. doi: 10.1186/s12913-022-07524-2 (PMC8862359; doi:10.1186/s12913-022-07524-2)
Supplement: Supplementary file 2 — Additional file 2. The MedHipPro Questionnaire [English paper version]. [file 12913_2022_7524_MOESM2_ESM.pdf]

Sykehuset i Vestfold

HELSE • SØR-ØST

QUESTIONNAIRE
PATIENT PATHWAY PHARMACIST

HIP FRACTURE PATIENTS

You have received this questionnaire because you are involved in the treatment of patients with hip fractures (femoral neck, pertrochanteric or subtrochanteric fracture).
<u>If you are not involved in hip fracture patients' treatment in any way, we ask you not to complete the questionnaire.</u>

A clinical pharmacist will be included in the hip fracture patient pathway. This is a possible action/measure to increase patient safety towards an increased patient safety and reduce the workload of healthcare professionals involved.

We want to obtain the best possible knowledge of the current status in regards to medication reconciliation, medication review and communication of medication information.

See more information on page 25, and personal privacy protection on the next page.

Yours faithfully,

Ben Tore Henriksen
Patient Pathway Pharmacist |
Clincal pharmacist | MSc Pharm
Vestfold Hospital Trust and
Department of Pharmaceutical Services,
Tønsberg Hospital Pharmacy
E-mail: Ben.Tore.Henriksen@sykehusapotekene.no
Office tel: +47 33 34 30 97

The questionnaire contains questions and statements. Please select your answer by checking the correct response option (tick box). If you wish to change answer after you have ticked a box, please fill the box that was incorrect, and tick the box that match your answer:

Incorrect option: ▩

Correct option: ☒

Please write free text response in the white area below the relevant question. If you wish to correct your response, strikethrough the part that you want to erase

Please write as clearly as possible.

It takes approximately 5-14 minutes to complete the questionnaire.
Your contribution is valuable! Thank you for taking the time to respond

DEADLINE FOR REPLYS: [INSERT DATE]

Personal privacy protection:

All information is treated confidentially. Analysis of identifiable questionnaires will only be done by Pharmacy master's student, [redacted] and Pharmacist [redacted] (the patient pathway pharmacist). Planned date of completion is 01 Apr 2020. Deidentified material is stored until the scheduled date of completion for processing personal data; 01 May 2028. After this date, the information is anonymised. The material is stored for future research.

The information registered about you will only be used as described for the purpose of the project. This consent is the legal basis for the processing of personal data. You have the right to access the information that is registered about you and the right to have any errors in the information corrected. You have the right to data portability, which means that you can receive the electronically registered personal information in a Microsoft Word document. You may, at any time, request all registered information to be deleted. You also have the right to send a complaint to the Privacy Ombudsman at the Norwegian Centre for Research Data (NSD):

E-mail: [redacted]
Telephone: [redacted]
Address: [redacted]

If you withdraw from the project, you can request that the collected information is deleted, unless the information has already been included in analyses or used in scientific publications. If you later wish to withdraw or have questions about the project, you may contact clinical pharmacist [redacted] on telephone [redacted] or by e-mail: [redacted]

This questionnaire is divided into four parts:

Part A: Background Information
Part B: Medication reconciliation / Obtaining Medication Information
Part C: Medication review (for physicians)
Part D: Medication list

Definitions are available under each section, and are described on page 24.

SECTION A – BACKGROUND INFORMATION

1. Are you a nurse or physician?
Please choose only one of the options

☐ Nurse

☐ Physician

Other (please specify): ____________________

2. Where are you employed?
Please choose only one of the options
If you are employed in several places, answer for the main position.

☐ General Practitioner's office
If you are a General Practitioner AND a physician in a nursing home, please answer for the main position

☐ District Nurse

☐ Care home

☐ Nursing home, for short term stay (with or without rehabilitation)

☐ Nursing home, for long term stay (with or without rehabilitation)

☐ Emergency care unit (Vestfold Hospital Trust)
House officers: choose emergency care unit (Vestfold Hospital Trust)

☐ Orthopaedic department (Vestfold Hospital Trust)

Other (please specify): ____________________

3. How many years of experience do you have as a doctor / nurse in total?

☐ Less than 1 year
☐ 1 year
☐ 2 years
☐ 3 years
☐ 4 years
☐ 5 years
☐ 6 years
☐ 7 years
☐ 8 years
☐ 9 years
☐ 10 years
☐ 11 years
☐ 12 years
☐ 13 years
☐ 14 years
☐ 15 years
☐ 16 years
☐ 17 years
☐ 18 years
☐ 19 years
☐ 20 years
☐ 21-30 years
☐ 31 years, or more

For employees in primary healthcare:

4. In which local authority are you employed?

- ☐ Larvik
- ☐ Sandefjord
- ☐ Tønsberg
- ☐ Færder
- ☐ Re
- ☐ Horten
- ☐ Holmestrand
- ☐ Sande
- ☐ Svelvik
- ☐ Other local authority not specified:
  Write in free text

5. What is your current work title / position?

Please choose only one of the options

PHYSICIAN:
Are you a specialist, or in training to become a specialist?

- ☐ Medical student with licence
- ☐ Foundation programme locum
  Temporary position, for example waiting for a Foundation programme position
- ☐ Foundation doctor (FY1 or FY2)
- ☐ Specialty registrar in orthopaedics
- ☐ Specialty registrar in hospital speciality (StR)
- ☐ Specialty registrar in general practice (GPST)
- ☐ Specialist in orthopaedics (Consultant)
- ☐ General Practitioner
- ☐ Other speciality registrar
- ☐ Specialist in other specialty (Consultant), please provide the specialty:__________________
- ☐ Neither in specialist training, nor a specialist
- ☐ Other (please specify): _________________________________

NURSE:

- ☐ Nurse with extended function (without further training/education)
- ☐ Nurse with extended function (with completed further training/education)
- ☐ Nurse (with further training/education)
  Independent of type of further training / specialisation
- ☐ Nurse (without further training/education)
- ☐ Other (please specify): _________________________________

Example of extended function: Professional development nurse. Some local authorities have other terms for an extended function. This option also applies to “Nurse-1” / “professional nurse” / “assistant nurse” / “inpatient nurse” / “assistant head of department” / “responsible nurse” / possibly other terms

If further training / education, please describe which:

For employees at Vestfold Hospital Trust:

6. What is your current work title / position?
Please choose only one of the options

PHYSICIAN:
Are you a specialist, or in training to become a specialist?

- ☐ Medical student with licence
- ☐ Foundation programme locum
  Temporary position, for example waiting for a Foundation programme position
- ☐ Foundation doctor (FY1 or FY2)
- ☐ Specialty registrar in orthopaedics
- ☐ Specialty registrar in hospital speciality (StR)
- ☐ Specialist in orthopaedics (Consultant)
- ☐ Specialist in other specialty (Consultant), please provide the specialty:__________________
- ☐ Other (please specify): _________________________________

NURSE:

- ☐ Nurse with extended function (without further training/education)
- ☐ Nurse with extended function (with completed further training/education)
- ☐ Nurse (without further training/education)
- ☐ Nurse (with further training/education)
  Independent of type of further training / specialisation
- ☐ Other (please specify): _________________________________

Example of extended function: Professional development nurse. Some local authoritiess have other terms for an extended function. This option also applies to "Nurse-1" / "professional nurse" / "assistant nurse" / "inpatient nurse" / "assistant head of department" / "responsible nurse / possibly other terms

If further training / education, please describe which:

PLEASE PICTURE THE FOLLOWING SCENARIO:

Select the scenario associated with your previously mentioned role

Time period: In the last three months

For employees in primary healthcare:

*For **General Practitioners (GPs)**:*
Picture that one of your patients has had a hip fracture and comes back to you after a stay in hospital and rehabilitation/nursing home. The patient uses three medications or more. You receive a medication list from the hospital and/or from the nursing home/rehabilitation institution. The medication list can be part of the discharge summary, information via the nursing service's electronic messages (PLO), or available separately. By performing a medication reconciliation, we here mean that you update the medication list in your system to make it is as accurate as possible. This involves considering the information in the medication list received. If there is no medication list/discharge summary, picture that you must actively obtain the information from the hospital/nursing home/rehabilitation.

*For **physicians at rehabilitation institutions** or (short term) **nursing home**:*
Picture that you receive a hip fracture patient admitted from the hospital. The patient uses three medications or more. You receive a medication list from the hospital. By performing a medication reconciliation, we here mean that you update the medication list in your system to make it in accordance with the information for the hospital. If there is no medication list, picture that you must actively obtain the information from the hospital. If you get the impression that the medication list is incorrect, or is incomplete, you must use one or more additional source to make it as correct as possible. If you are not present every day at the rehabilitation institution, answer only for the days you are there physically.

*For **physicians at** (long term) **nursing home**:*
Picture that you receive a hip fracture patient admitted to your nursing home. The patient uses three medications or more. You receive a medication list from the hospital. By performing a medication reconciliation, we here mean that you update the medication list in your system to make it in accordance with the information from the setting the patient was transferred from (rehabilitation, hospital, etc). If there is no medication list, picture that you must actively obtain the information from the hospital. If you get the impression that the medication list is incorrect, or is incomplete, you must use one or more additional source to make it as correct as possible. If you are not present every day at the nursing home, answer only for the days you are there physically.

*For **nurses** in **nursing homes** and/or **rehabilitation**:*
Picture that you receive a hip fracture patient admitted to your nursing home/rehabilitation institution. The patient uses three medications or more. You are delegated, or initiate on your own, the task of obtaining a medication list from another setting (see definition).

*For **district nurses**:*
Picture that you have a patient who had a hip fracture and needs help with the administration of medication partially or fully by you. The patient uses three medications or more. A medication list follows the patient from the hospital and/or the rehabilitation institution. You are delegated, or initiate on your own, the task of obtaining a medication list from another setting (e.g. general practitioner).

PLEASE PICTURE THE FOLLOWING SCENARIO:

Select the scenario associated with your previously mentioned role

Time period: In the last three months

For employees at Vestfold Hospital Trust:

For ***nurses*** *in* ***emergency care unit****:*
Picture that you receive a patient admitted with a hip fracture. The patient uses three medications or more. You are delegated, or initiate on your own, the task of obtaining a medication list from another setting (see definition).

For ***nurses*** *in* ***orthopaedic department****:*
Picture that you receive a patient admitted with a hip fracture. The patient uses three medications or more. You are delegated, or initiate on your own, to perform medication reconciliation. By performing a medication reconciliation, we here mean that you obtain information from the sources required for the medication list to represent what the patient actually uses. Relevant sources are the General Practitioner (GP), district nurses, digital summary of care record, conversation with patient, or similar.

For ***physicians****:*
Picture that you receive a patient admitted with a hip fracture. The patient uses three medications or more. By performing a medication reconciliation, we here mean that you obtain information from the sources required for the medication list to represent what the patient actually uses. Relevant sources are the General Practitioner (GP), district nurses, digital summary of care record, conversation with patient, or similar.

THE FOLLOWING SECTION IS FOR PHYSICIANS AND NURSES
- EXCEPT NURSES IN EMERGENCY CARE UNIT, PLEASE SEE SECTION C -
Section B – Medication reconciliation

Do you perform medication reconciliation?

Answer yes if you occasionally perform medication reconciliation or try as best you can when you have the opportunity

☐ YES ☐ NO (skip to section D)

☐ NURSES IN EMERGENCY CARE UNIT:
Skip to «SECTION C – OBTAINING MEDICATION LIST»

For all nurses (except nurses in emergency care unit):
Answer yes to the fact that you perform medication reconciliation if you obtain medication lists and compare these in collaboration with the patient, where applicable. There may be a doctor who signs for the completion of medication reconciliation and prescribes based on your input. Answer yes if you occasionally perform medication reconciliation or try as best you can when you have the opportunity.
If you never perform medication reconciliation, skip to section C or D.

Medication reconciliation / reconciliation:
Medication reconciliation is a method where healthcare personnel in collaboration with the patient secures **complete information** of all medication used by the patient. This entails obtaining the correct medication list.

Medication list
An updated list of all medications the patient uses [Norwegian abbreviation: 'LIB-list']. Examples of medication lists are the GP's list, the discharge summary and the list from the district nurses. From experience, on some occasions, the medication list is sent separately. However, it should be part of the discharge summary when patients are discharged from hospital. For patients in rehabilitation, short-term or long-term stay in a nursing home, this means a medication list at discharge that is sent to the next institution, district nurse and/or GP, as well as to the patient when possible. Medication chart is not considered a medication list. This is specified in the question if relevant.

Medication information
By medication information we hereby imply the medication list and any information regarding planned changes in dosage regimen, discontinuation of treatment, addition of treatment, follow-up, blood tests or similar.

We would like to remind you that the questionnaire is regarding hip fracture patients only. If hip fracture patients does not differ from other patients, please picture generally for all patients you are involved in. Picture the last three months.

	Please respond to the following statements	Strongly disagree	Disagree	Neither agree, nor disagree	Agree	Strongly agree
1	*For all respondents, except emergency care unit:*<br>My impression is that most of my patients have a correct medication list	☐	☐	☐	☐	☐
2	*For all respondents:*<br>I obtain as much information as I can when I personally perform medication reconciliation, so I am confident that it is as accurate as possible<br>*Examples: Patient/carer, district nurse, GP's list, electronic prescription database, multi-dose drug dispensing chart, digital summary of care record. Preferably a combination of these.*	☐	☐	☐	☐	☐

We would like to remind you that the questionnaire is regarding hip fracture patients only. If hip fracture patients does not differ from other patients, please picture generally for all patients you are involved in. Picture the last three months.

	**Please respond to the following statements**	Strongly disagree	Disagree	Neither agree, nor disagree	Agree	Strongly agree
3	*For everyone, except GP and district nurses:* It happens often that the patient comes to me/us without a medication list	☐	☐	☐	☐	☐
4	*For GPs:* It happens often that the patient comes back to me without a medication list	☐	☐	☐	☐	☐
5	*For GPs:* I always receive correct medication information from the hospital	☐	☐	☐	☐	☐
6	*For GPs:* I always receive correct medication information after a nursing home stay or rehabilitation	☐	☐	☐	☐	☐
7	*For physicians at nursing homes/rehabilitation:* The medication information I receive at admission is always correct	☐	☐	☐	☐	☐
8	*For physicians at hospital and nursing homes/rehabilitation:* I always delegate the **medication reconciliation** task if I, personally, is unable to perform medication reconciliation	☐	☐	☐	☐	☐
9	*For orthopaedic department:* I always trust that the medication chart coming from the emergency care unit is correct even if medication reconciliation is not documented anywhere	☐	☐	☐	☐	☐
10	*For orthopaedic department:* I always trust that the medication chart coming from the emergency care unit is correct when medication reconciliation is documented somewhere (for example on the dedicated stick-on label on the medication chart or in the patient's admission summary [in the electronic medical journal])	☐	☐	☐	☐	☐

Questions

For hospital physicians and nurses [at Vestfold Hospital Trust]:

11. Once you personally have performed medication reconciliation, do you document it?

The fact that the medication is prescribed is not documentation of medication reconciliation. By documenting we imply that it is documented in a medical record or similar. At the hospital, the dedicated medication reconciliation stick-on label is included as documentation.

Please choose only one response option.

- ☐ Yes, always (approximately 90 % of the cases, or more)
- ☐ Yes, often (app. 60-89 % of the cases)
- ☐ Yes, sometimes (app. 30-59 % of the cases)
- ☐ Yes, but rarely (app. 1-29 % of the cases)
- ☐ Never (skip to question 15)

We would like to remind you that the questionnaire is regarding hip fracture patients only. If hip fracture patients does not differ from other patients, please picture generally for all patients you are involved in. Picture the last three months.

Questions

For hospital physicians and nurses [at Vestfold Hospital Trust]:

12. If you document the completion of medication reconciliation; where do you document it?

More than one option possible

☐ Medical record

☐ The dedicated medication reconciliation stick-on label, on the hospital medication chart

Another place – please specify:

For hospital physicians and nurses [at Vestfold Hospital Trust]:

13. What prevents you from being able to document a medication reconciliation?

More than one option possible

☐ I do not see the need<br>☐ I am not aware that it should be done / I do not think about it<br>☐ Not possible electronically<br>☐ The medication chart lacks the stick-on label<br>☐ Lack of time	Other:

For hospital physicians and nurses [at Vestfold Hospital Trust]:

14. What can been done for you to document medication reconciliations more often?

More than one option possible

☐ Improved electronic systems<br>☐ More time for each patient<br>☐ Regular multidisciplinary meetings (more often)<br>☐ Raised awareness<br>☐ Reminders<br>☐ More medication charts have the dedicated stick-on label	Annet:

For all:<br>**15. Which actions would you suggest would allow you to spend less time performing a medication reconciliation?**
For all:<br>**16. Other comments regarding medication reconciliation?**

THE FOLLOWING SECTION IS FOR NURSES IN EMERGENCY CARE UNIT ONLY

Section C – Obtaining medication list

In the upper right corner, on the back side of the new emergency care chart, there are questions that says:

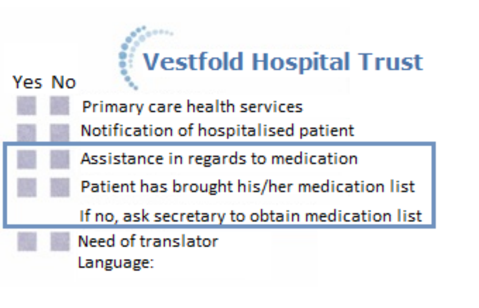

Vestfold Hospital Trust

Yes	No	
☐	☐	Primary care health services
☐	☐	Notification of hospitalised patient
☐	☐	Assistance in regards to medication
☐	☐	Patient has brought his/her medication list
		If no, ask secretary to obtain medication list
☐	☐	Need of translator
		Language:

The next questions are regarding the questions above.

Medication list
An updated list of all medications the patient uses [Norwegian abbreviation: 'LIB-list']. Examples of medication lists are the GP's list, the discharge summary and the list from the district nurses. From experience, on some occasions the medication list is sent separately, but the medication list should be part of the discharge summary when patients are discharged from hospital. For patients in rehabilitation, short-term or long-term stay in a nursing home, this means a medication list at discharge that is sent to the next institution, district nurses and/or GP, as well as to the patient when possible. Medication chart is not considered a medication list. This is specified in the question if relevant.

Obtaining medication lists
By obtaining medication lists we hereby imply obtaining one or more lists of current medication. It does not matter if there is a list of medication already present or if there is no list present. The medication list(s) you obtain must be used to perform medication reconciliation. You can give this medication list to a physician directly, or personally reconcile the medication list before giving information to a physician. Relevant sources are GPs, district nursing, electronic medical records, conversations with patients / relatives or similar.

We would like to remind you that the questionnaire is regarding hip fracture patients only. If hip fracture patients does not differ from other patients, please picture generally for all patients you are involved in. Picture the last three months.

	**Please respond to the following statements**	Strongly disagree	Disagree	Neither agree, nor disagree	Agree	Strongly agree
1	If the patient has assistance in regards to administration/handling of medication I always ask the secretary to **obtain a medication list**	☐	☐	☐	☐	☐

Questions

2. In the instances where you do not ask the secretary to <u>obtain a medication list</u>, why do you not ask?
Imagine a situation where it typically is natural to ask - for example if the patient has district nurses responsible for medication management
More than one option possible

☐ I do not see the need
☐ I am not aware that it should be done / I do not think about it
☐ Too many tasks to perform simultaneously
☐ It is not arranged for
☐ Difficult to remember if I have asked or not
☐ Not sure if others have asked the secretary / assume that others have asked the secretary
☐ Cannot return to the patient (allocated to another patient / goes off duty or similar)

Annet:

We would like to remind you that the questionnaire is regarding hip fracture patients only. If hip fracture patients does not differ from other patients, please picture generally for all patients you are involved in. Picture the last three months.

Questions

3. What can been done for you to ask the secretary if he/she can obtain a medication list more often?
Imagine a situation where it typically is natural to ask - for example if the patient has district nurses responsible for medication management
More than one option possible

☐ More time for each patient<br>☐ Less tasks to perform simultaneously<br>☐ Raised awareness<br>☐ Reminder	Annet:

4. When you have personally asked the secretary to obtain a medication list, do you document it in the emergency care chart?
Please choose only one option

☐ Yes, always (approximately 90 % of the cases, or more)
☐ Yes, often (app. 60-89 % of the cases)
☐ Yes, sometimes (app. 30-59 % of the cases)
☐ Yes, but rarely (app. 1-29 % of the cases)
☐ Never

5. In the instances where you do not document, why do you not document?
Picture a scenario where you have asked the secretary to obtain a medication list, but you have not documented it
More than one option possible

☐ I am not aware that it should be done / I do not think about it<br>☐ Too many tasks to perform simultaneously<br>☐ It is not arranged for<br>☐ Difficult to remember if I have asked or not<br>☐ Not sure if others have asked the secretary / assume that others have asked the secretary<br>☐ Cannot return to the patient (allocated to another patient / goes off duty or similar)	Other:

We would like to remind you that the questionnaire is regarding hip fracture patients only. If hip fracture patients does not differ from other patients, please picture generally for all patients you are involved in. Picture the last three months.

**Questions**	
**6. What could be done for you to document?**<br>*More than one option possible*	
☐ Improved electronic systems<br><br>☐ More time for each patient<br><br>☐ Raised awareness<br><br>☐ Have the questions on the fast track check-list<br><br>☐ Reminder<br><br>☐ Place the questions* elsewhere on the emergency care chart	Other:

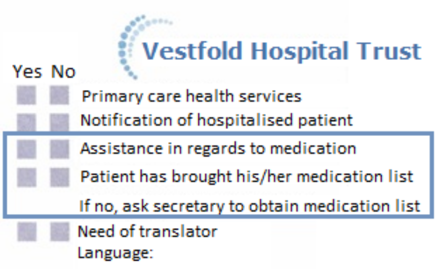

* In the upper right corner, on the back side of the new emergency care chart, there are questions that says:

We would like to remind you that the questionnaire is regarding hip fracture patients only. If hip fracture patients does not differ from other patients, please picture generally for all patients you are involved in. Picture the last three months.

**5. Other comments regarding <u>obtaining medication lists?</u>**

THE FOLLOWING SECTION IS FOR PHYSICIANS ONLY

Part D: Medication review

Do you perform a medication review?

☐ YES ☐ NO (skip to section E)

☐ House officers working in emergency care unit:
Skip to «Section E – Medication list»

Answer yes to the fact that you perform medication reviews if you occasionally perform medication reviews, or try as best you can when you have the opportunity.

If you never perform medication reviews, skip to section E.

Medication review
A medication review is a systematic process to assure quality of the individual patient's use of medication, in order to ensure efficacy and safety. Examples of content in a medication review; correct indication and/or dosage, drug-drug interactions, side effects and inappropriate drug treatment.

The medication review can be done by the physician treating the patient, or in multidisciplinary team where the physician treating the patient is part of the team. The physician is responsible for the final decision on further pharmacologic treatment for the patient. Medication reviews are based on relevant clinical information.

We would like to remind you that the questionnaire is regarding hip fracture patients only. If hip fracture patients does not differ from other patients, please picture generally for all patients you are involved in. Picture the last three months.

	**Please respond to the following statements**	Strongly disagree	Disagree	Neither agree, nor disagree	Agree	Strongly agree
**1**	*For all physicians:*<br>More of my patients need medication review	☐	☐	☐	☐	☐
**2**	*For all physicians:*<br>I do not have the opportunity to perform (more) drug reviews myself	☐	☐	☐	☐	☐
**3**	*For all physicians:*<br>I want someone else to perform medication reviews<br>*By the term "someone else" we hereby imply qualified health personnel, such as another physician with the same specialty, another physician with another specialty, clinical pharmacist or similar.*	☐	☐	☐	☐	☐

4. Comments:

We would like to remind you that the questionnaire is regarding hip fracture patients only. If hip fracture patients does not differ from other patients, please picture generally for all patients you are involved in. Picture the last three months.

5. What do you include/consider in a medication review?
More than one option possible:

☐ I never perform medication reviews (skip to question 14)

☐ Monitoring of medication (i.e. medication where laboratory tests must be taken to assess effect)

☐ Medication management, administration and compliance/adherence

☐ Inappropriate medication for elderly
Some physicians use lists of inappropriate medication for elderly, such as the STOPP-list, Beers criteria, NorGEP criteria, or similar, but this is not a requirement to tick

☐ Discontinuing treatment without indication

☐ Optimising pharmacological treatment of all diagnosis/indications

☐ Clinically relevant medication interactions

☐ Individual assessment of all dosages (dosage adjusted to organ function (reduced renal function and/or liver function), drug allergies, etc.)

☐ Time-limited medication use (correct medication, correct dosage, correct duration)

☐ Medication-induced side-effect, symptom or altered laboratory test results

Other considerations (please specify):

6. Are you currently performing a comprehensive medication review where you also consider medication prescribed by other physicians?
Please choose only one option:

☐ Yes, every patients receive medication reviews (approximately 90 % of patients, or more)

☐ Yes, most patients receive medication reviews (app. 60-89 % of patients)

☐ Yes, some patients receive medication reviews (app. 30-59 % of patients)

☐ Yes, a few patients receive medication reviews (app. 1-29 % of patients)

☐ No, this type of medication review is not performed (skip to question 10)

We would like to remind you that the questionnaire is regarding hip fracture patients only. If hip fracture patients does not differ from other patients, please picture generally for all patients you are involved in. Picture the last three months.

7. Out of those patients who receives medication reviews, how often do they receive it?
Estimate mean:
Please choose only one option.

- ☐ Once per week
- ☐ Twice per week
- ☐ 3 times per week
- ☐ 4 times per week
- ☐ 5 times per week
- ☐ 6 times per week
- ☐ Every day
- ☐ Approximately biweekly
- ☐ App. once a month
- ☐ App. 7-11 times per year
- ☐ App. 2-6 times per year
- ☐ App. once a year
- ☐ Less than once a year
- ☐ Only when there are changes made to the treatment regimen
- ☐ At each consultation/visit, regardless of whether there are changes to the treatment regimen
- ☐ At each consultation/visit, but only if there are changes made to the treatment regimen
- ☐ Completely random

Other option not specified above:

For physicians in rehabilitation and/or nursing homes
8. Estimate the proportions of medication reviews that are discussed in a multidisciplinary forum
Please choose only one option

- ☐ Approximately 90 % of the medication reviews, or more
- ☐ App. 60-89 % of the medication reviews
- ☐ App. 30-59 % of the medication reviews
- ☐ App. 1-29 % of the medication reviews
- ☐ None (skip to question 11)

9. In which setting?
More than one option possible.

- ☐ Pre-ward round meetings
- ☐ Care team meetings
- ☐ Multidisciplinary meetings

Other setting/meeting:

10. Please mention the occupational groups you know attend most meetings:

- ☐ Physician
- ☐ Nurse
- ☐ Pharmacist
- ☐ Physiotherapist
- ☐ Social worker
- ☐ Social educator
- ☐ Other:

We would like to remind you that the questionnaire is regarding hip fracture patients only. If hip fracture patients does not differ from other patients, please picture generally for all patients you are involved in. Picture the last three months.

For physicians at orthopaedic department:
11. Estimate the proportion of hip fracture patients who are scheduled for a review at the weekly ortho-geriatric meeting:
Please choose only one option.

- ☐ Everyone (Approximately 90 % of patients or more)
- ☐ Most patients (App. 60-89 % of patients
- ☐ Some (App. 30-59 % of patients)
- ☐ A few (App. 1-29 % of patients)
- ☐ None

For physicians at orthopaedic department:
12. Are medication reviews documented?
This includes both **geriatric examinations**, **ortho-geriatric meetings** and other independent medication reviews:
Please choose only one option.

- ☐ Yes, always (approximately 90 % of medication reviews, or more)
- ☐ Yes, often (app. 60-89 % of medication reviews)
- ☐ Yes, sometimes (app. 30-59 % of medication reviews)
- ☐ Yes, but rarely (app. 1-29 % of medication reviews)
- ☐ No, I do not document (skip to question 11)

13. If yes, where do you document?
More than one option possible

- ☐ Medical record
- ☐ On the medication chart

**Elsewhere (please specify):**	**Other comments regarding documenation:**

14. Which actions would you suggest would allow more patients to receive a medication review where you also consider medications prescribed by other physicians?
More than one option possible

- ☐ Better payment (for GPs only)
- ☐ Better electronic/digital systems
- ☐ More time for each patient
- ☐ Regular multidisciplinary meetings (more often)
- ☐ Getting reminders that it should be done / request from others
- ☐ Increased knowledge (such as courses)

Other:

We would like to remind you that the questionnaire is regarding hip fracture patients only. If hip fracture patients does not differ from other patients, please picture generally for all patients you are involved in. Picture the last three months.

15. Other comments regarding medication review?

THE FOLLOWING SECTION IS FOR ALL PHYSICIANS

Section E: Medication list

Medication list
An updated list of all medications the patient uses [Norwegian abbreviation: 'LIB-list']. Examples of medication lists are the GP's list, the discharge summary and the list from the district nurses. From experience, on some occasions, the medication list is sent separately. However, it should be part of the discharge summary when patients are discharged from hospital. For patients in rehabilitation, short-term or long-term stay in a nursing home, this means a medication list at discharge that is sent to the next institution, district nurses and/or GP, as well as to the patient when possible. Medication chart is not considered a medication list. This is specified in the question if relevant.

We would like to remind you that the questionnaire is regarding hip fracture patients only. If hip fracture patients does not differ from other patients, please picture generally for all patients you are involved in. Picture the last three months.

	**Please respond to the following statements**	Strongly disagree	Disagree	Neither agree, nor disagree	Agree	Strongly agree
1	*For everyone:*<br>It happens often that the patient comes to me/us without a medication list	☐	☐	☐	☐	☐
2	*For orthopaedic department. Skip this question if you have answered to section B:*<br>I always trust that the medication chart coming from the emergency care unit is correct even if medication reconciliation is <u>not documented</u> anywhere	☐	☐	☐	☐	☐
3	*For orthopaedic department. Skip this question if you have answered to section B:*<br>I always trust that the medication chart coming from the emergency care unit is correct when medication reconciliation <u>is documented</u> somewhere (for example on the dedicated stick-on label on the medication chart or in the patient's admission summary [in the electronic medical journal])	☐	☐	☐	☐	☐
4	*For General Practitioners:*<br>The hospital always requests an updated medication list when a patient is admitted to hospital with a hip fracture	☐	☐	☐	☐	☐
5	*For General Practitioners:*<br><u>When you send a medication list to the hospital:</u><br>I always spend time double checking that the medication list is as accurate as possible<br>*E.g. comparing (or double-checking) the medication list with the electronic medication database or the last medical record containing medication information*	☐	☐	☐	☐	☐
6	*For physicians in rehabilitation institution and/or nursing home:*<br><u>In the discharge report:</u><br>I always write a complete medication list when I am responsible for the discharge of a patient	☐	☐	☐	☐	☐
7	*For physicians in orthopaedic ward, rehabilitation institutions and/or nursing home:*<br><u>In the discharge summary and/or discharge report:</u><br>I always spend time double checking that the medication list is as accurate as possible<br>*E.g. comparing (or double-checking) the medication list with the medication chart / kardex*	☐	☐	☐	☐	☐

We would like to remind you that the questionnaire is regarding hip fracture patients only. If hip fracture patients does not differ from other patients, please picture generally for all patients you are involved in. Picture the last three months.

Questions

For General Practitioners:

8. If changes in the medication regimen: Do you write a complete medication list for the one who administers medication?

Please choose only one response option.

- ☐ Yes, always (approximately 90 % of the cases, or more)
- ☐ Yes, often (app. 60-89 % of the cases)
- ☐ Yes, sometimes (app. 30-59 % of the cases)
- ☐ Yes, but rarely (app. 1-29 % of the cases)
- ☐ No

For physicians at orthopaedic department:

9. How often do you write the medication list in the discharge summary without following the local procedure [i.e. the “NEK”-template at Vestfold Hospital Trust]?

Please choose only one response option.

- ☐ Always (approximately 90 % of the cases, or more)
- ☐ Often (app. 60-89 % of the cases)
- ☐ Sometimes (app. 30-59 % of the cases)
- ☐ Rarely (app. 1-29 % of the cases)
- ☐ Never

For physicians at orthopaedic department:

10. How often do you write the medication list in the discharge summary following the local procedure [i.e. the “NEK”-template at Vestfold Hospital Trust]?

Please choose only one response option.

- ☐ Always (approximately 90 % of the cases, or more)
- ☐ Often (app. 60-89 % of the cases)
- ☐ Sometimes (app. 30-59 % of the cases)
- ☐ Rarely (app. 1-29 % of the cases)
- ☐ Never

For physicians at orthopaedic department:

11. How often do discharge a patient without including (?) a medication list in the discharge summary?

Please choose only one response option.

- ☐ Always (approximately 90 % of the cases, or more)
- ☐ Often (app. 60-89 % of the cases)
- ☐ Sometimes (app. 30-59 % of the cases)
- ☐ Rarely (app. 1-29 % of the cases)
- ☐ Never

We would like to remind you that the questionnaire is regarding hip fracture patients only. If hip fracture patients does not differ from other patients, please picture generally for all patients you are involved in. Picture the last three months.

For physicians at nursing homes:

12. At discharge: Do you write a complete medication list for the one who administers medication?

Please choose only one response option.

- ☐ Always (approximately 90 % of the cases, or more)
- ☐ Often (app. 60-89 % of the cases)
- ☐ Sometimes (app. 30-59 % of the cases)
- ☐ Rarely (app. 1-29 % of the cases)
- ☐ Never

For physicians at orthopaedic department:

13. How much time would you estimate it takes (on average) for you to write the medication part of the discharge summary if you do not follow the local procedure [i.e. the "NEK"-template at Vestfold Hospital Trust]?

Please choose only one response option.

- ☐ < 1 minute
- ☐ 1-2 minutes
- ☐ 3-5 minutes
- ☐ 6-10 minutes
- ☐ 11-20 minutes
- ☐ 21 minutes or more
- ☐ I always follow the local procedure
- ☐ I do not write a medication list

For physicians at orthopaedic department:

14. How much time would you estimate it takes (on average) for you to write the medication part of the discharge summary if you follow the local procedure [i.e. the "NEK"-template at Vestfold Hospital Trust]?

Please choose only one response option.

- ☐ < 1 minute
- ☐ 1-2 minutes
- ☐ 3-5 minutes
- ☐ 6-10 minutes
- ☐ 11-20 minutes
- ☐ 21 minutes or more
- ☐ I never follow the local procedure
- ☐ I do not write a medication list

For physicians at the Emergency Care Unit:

15. How much time would you estimate it takes (on average) for you to write the medication part of the admission summary medical record?

Please choose only one response option.

- ☐ < 1 minute
- ☐ 1-2 minutes
- ☐ 3-5 minutes
- ☐ 6-10 minutes
- ☐ 11-20 minutes
- ☐ 21 minutes or more
- ☐ I do not write a medication list

We would like to remind you that the questionnaire is regarding hip fracture patients only. If hip fracture patients does not differ from other patients, please picture generally for all patients you are involved in. Picture the last three months.

For physicians at orthopaedic department:

16. Do you provide verbal information to patients and/or relatives about changes in the drug regimen?

You can ignore this for those patients who do not administer medication themselves

- ☐ Yes, always (approximately 90 % of the cases, or more)
- ☐ Yes, often (app. 60-89 % of the cases)
- ☐ Yes, sometimes (app. 30-59 % of the cases)
- ☐ Yes, but rarely (app. 1-29 % of the cases)
- ☐ No

For the Emergency Care Unit:

17. When the patient is transferred from the Emergency Care Unit to the Orthopedic section: What could be improved in regards to communication of medication information?

For nurses at the orthopaedic department:

18. When patients are transferred from the Emergency Care Unit: What could be improved in regards to communication of medication information?

We would like to remind you that the questionnaire is regarding hip fracture patients only. If hip fracture patients does not differ from other patients, please picture generally for all patients you are involved in. Picture the last three months.

For all (except nurses at Orthopaedic section):
19. When the patient is transferred from the previous care setting or institution:
What could be improved in regards to communication of medication information?

Examples of transfer between care setting / institution:

- **Hospital:** At admission
- **Nursing home / rehabilitation:** At admission after hospital stay
- **GP:** After hip fracture, with information from hospital / nursing home / rehabilitation
- **District nurses:** To the home from a hospital and/or institution (nursing home and/or rehabilitation)

For all (except Emergency Care Unit):
20. When the patient is transferred to the next care setting or institution:
What could be improved in regards to communication of medication information?

Examples of transfer between care level / institution:

- **Hospital:** At discharge to rehabilitation / nursing home / home (with or without district nurses)
- **Nursing home / rehabilitation:** At discharge to home / other institution with information to General Practitioner
- **GP:** At admission to hospital
- **District nurses:** At admission to hospital

ENDING
**Other comments regarding pharmacological treatment of hip fracture patients?**

Background questions	
**1. Gender**	**2. Year of birth**
☐ **Female**<br>☐ **Male**	

Desire for further participation
**Some people may be contacted to further contribute to the project (new questionnaire, interview, or similar). If you are interested in this, please enter your personal details below.**
All information is treated confidentially. Analysis of identifiable questionnaires will only be processed by [redacted] and the patient pathway pharmacist. See additional information on privacy on page 2.
**1. Name**
**2. E-mail address**
**3. Telephone number**

DEFINITIONS OF TERMS USED IN THE QUESTIONNAIRE

The last five terms are repeated in the start of the respective section.

Patient Pathway Pharmacist
A clinical pharmacist included in an entire patient pathway, regardless of department affiliation. The patient pathway pharmacist will follow a patient group through a patient pathway and perform the following tasks: drug reconciliation, medication review, ensure optimisation of pharmacological treatment related to the present situation and assist in the transfer of key information related to treatment.

Hip fracture patients
Patients with fracture in the proximal femur bone: femoral neck, pertrochanteric or subtrochanteric fractures.

Medication review
A medication review is a systematic process to assure quality of the individual patient's use of medication, in order to ensure efficacy and safety. Examples of content in a medication review; correct indication and/or dosage, drug-drug interactions, side effects and inappropriate drug treatment.

The medication review can be done by the physician treating the patient, or in mulitdisciplinary team where the physician treating the patient is part of the team. The physician is responsible for the final decision on further pharmacologic treatment for the patient. Medication reviews are based on relevant clinical information.

Medication list
An updated list of all medications the patient uses [Norwegian abbreviation: 'LIB-list']. Examples of medication lists are the GP's list, the discharge summary and the list from the district nurses. From experience, on some occasions, the medication list is sent separately. However, it should be part of the discharge summary when patients are discharged from hospital. For patients in rehabilitation, short-term or long-term stay in a nursing home, this means a medication list at discharge that is sent to the next institution, district nurse and/or GP, as well as to the patient when possible. Medication chart is not considered a medication list. This is specified in the question if relevant.

Medication reconciliation / reconciliation:
Medication reconciliation is a method where healthcare personnel in collaboration with the patient secures **complete information** of all medication used by the patient. This entails obtaining the correct medication list.

Obtaining medication lists
By obtaining medication lists we hereby imply obtaining one or more lists of current medication. It does not matter if there is a list of medication already present or if there is no list present. The medication list(s) you obtain must be used to perform the medication reconciliation. You can give this medication list to a physician directly, or personally reconcile the medication list before giving information to a physician. Relevant sources are GPs, district nurses, electronic medical records, conversations with patients / relatives or similar.

Medication information
By medication information we hereby imply the medication list and any information regarding planned changes in dosage regimen, discontinuation of treatment, addition of treatment, follow-up, blood tests or similar.

GENERAL INFORMATION REGARDING THE PROJECT:
Patient pathway pharmacist
Drug optimisation for hip fracture patients – facilitating a safe patient handover: A descriptive study

Safe medication management is a global challenge and a strategic focus area. Medication errors in the patient pathways constitutes a significant patient safety risk. In the United States, medical errors are the third highest cause of death in society. Despite laws, regulations and a number of national measures ([Norwegian] Patient Safety Program), as well as local procedures, guidelines, audits and supervision, one does not succeed in ensuring that the patients are prescribed and use the correct medication. There are challenges with the communication between the various medical record systems, both in primary and secondary healthcare. This results, among other things, in the lack or limited transfer of medication information when the patient is admitted to hospital. In addition, patient handovers; for example, hospitals to nursing homes, nursing homes to district nurses/GP's, etc., result in incorrect medication lists. In addition to the correct prescribing and use of medicines, there is thus great challenges with the transfer of medication information and consequently discrepancies in medication lists.

A dedicated pharmacist that follows the patient pathway (Patient Pathway Pharmacist) can create a smoother transition from the primary healthcare, into the patient pathway in secondary healthcare and handover back to the primary healthcare. This descriptive project is meant to be a quality-improving initiativ. The patient's pharmacological treatment will be assessed on a continuous basis, with medication reconciliation and medication review performed and key medication information communicated. All steps in the patient pathway are covered with optimal pharmacological follow-up and thus increased patient safety. This is a measure to improve the above challenges.

The project will assess the Patient Pathway Pharmacist's position and specific tasks in the hip fracture patient pathway at Vestfold Hospital Trust, and the patient pathway extended to primary healthcare. It will highlight areas where the pharmacist contributes to increased patient safety and improve communication in transitions across care levels based on drug optimisation throughout the pathway. In addition, evaluate perceived benefits of the Patient Pathway Pharmacist via qualitative methods.

The comparing group is baseline data, which in this case is an investigation of the last 50 patients' medical records.
An estimate of patients receiving the intervention is 60 patients. Start-up is estimated to the third or fourth quarter of 2018, and with duration of the first or second quarter of 2019.

Do not hesitate to contact us with questions or requesting more information. See contact details on the first page.

Primary endpoints
1) Discharge summary score
2) Medication reconciliation score
3) Number of discharge summaries written in accordance with local procedure
4) Number of inappropriate medication for the elderly at discharge

Qualitative endpoints:

1) Healthcare professionals experience of the current medication-related treatment of hip fracture patients
2) Experienced advantages and disadvanted with a Patient Pathway Pharmacist

Your contribution is valuable! Thank you for taking the time to complete the questionnaire.

Please return the questionnaire to [redacted] *in the attached envelope.*

Are you employed at the hospital, please return the questionnaire by internal mail system and label the envolope with:

«[redacted] – *Questionnaire – CONFIENTIAL»*
